# Change in Leukocyte Telomere Length Predicts Mortality in Patients with Stable Coronary Heart Disease from the Heart and Soul Study

**DOI:** 10.1371/journal.pone.0160748

**Published:** 2016-10-26

**Authors:** Sarah E. Goglin, Ramin Farzaneh-Far, Elissa S. Epel, Jue Lin, Elizabeth H. Blackburn, Mary A. Whooley

**Affiliations:** 1 Department of Medicine, University of California San Francisco, San Francisco, CA, 94143, United States of America; 2 Department of Psychiatry, University of California San Francisco, San Francisco, CA, 94143, United States of America; 3 Department of Biochemistry and Biophysics, UCSF, San Francisco, CA, 94143, United States of America; 4 Veterans Affairs Medical Center, San Francisco, CA, 94121, United States of America; Universite de Sherbrooke Faculte de medecine et des sciences de la sante, CANADA

## Abstract

**Background:**

Short telomere length independently predicts mortality in patients with coronary heart disease. Whether 5-year change in telomere length predicts subsequent mortality in patients with coronary heart disease has not been evaluated.

**Methods:**

In a prospective cohort study of 608 individuals with stable coronary artery disease, we measured leukocyte telomere length at baseline and after five years of follow-up. We divided the sample into tertiles of telomere change: shortened, maintained or lengthened. We used Cox survival models to evaluate 5-year change in telomere length as a predictor of mortality.

**Results:**

During an average of 4.2 years follow-up, there were 149 deaths. Change in telomere length was inversely predictive of all-cause mortality. Using the continuous variable of telomere length change, each standard deviation (325 base pair) greater increase in telomere length was associated with a 24% reduction in mortality (HR 0.76, 95% CI 0.61–0.94; p = 0.01), adjusted for age, sex, waist to hip ratio, exercise capacity, LV ejection fraction, serum creatinine, and year 5 telomere length. Mortality occurred in 39% (79/203) of patients who experienced telomere shortening, 22% (45/203) of patients whose telomere length was maintained, and 12% (25/202) of patients who experienced telomere lengthening (p<0.001). As compared with patients whose telomere length was maintained, those who experienced telomere lengthening were 56% less likely to die (HR 0.44, 95% CI, 0.23–0.87).

**Conclusions:**

In patients with coronary heart disease, an increase in leukocyte telomere length over 5 years is associated with decreased mortality.

## Significance

Leukocyte telomere length may serve as a marker of biological aging and predictor of mortality. Whether 5-year change in telomere length predicts subsequent mortality has not been evaluated. In a longitudinal study of 608 patients who were followed for 9.2 years, we found that leukocyte telomere shortening during the first five years was an independent predictor of mortality during the subsequent 4.2 years. Every 325 base pair increase in telomere length during the first 5 years was associated with 24% lower mortality during follow-up. This is the first study to show that 5-year change in telomere length predicts subsequent mortality.

## Introduction

Telomeres are tandem DNA repeat sequences at the ends of eukaryotic chromosomes that protect genomic information during mitosis[1]. During cell division, DNA polymerase cannot fully replicate the 3’ end of linear DNA, resulting in a progressive loss of telomere repeats[2]. After a critical degree of telomere shortening, cells can undergo transcriptional profile alterations, lose the ability to replicate and cease dividing (cellular senescence), and may undergo apoptosis[3]. Human telomere length is influenced by both genetic and environmental factors[4]. These observations have led to an increasing interest in telomere maintenance as the possible basis for a “biological clock,” as initially proposed on theoretical grounds by Olonikov in 1973, which integrates the cumulative lifetime burden of genetic factors and environmental stressors independently of chronological age[5].

The strongest evidence that cellular aging, as represented by short telomeres, might be associated with human aging in populations (as well as in carriers of mutations known to directly impair telomere maintenance [6] has primarily been derived from cross sectional studies. Prior studies have shown an independent association between short telomere length and cardiovascular events, including myocardial infarction[7], congestive heart failure[8], stroke[9] and death[10, 11]. However, little is known about the dynamic change and regulation of telomere length over time, and their consequences. One longitudinal study of 236 healthy men found that leukocyte telomere length shortening was a predictor of subsequent cardiovascular mortality[12]. However, no studies have examined whether change in leukocyte telomere length predicts mortality in patients with coronary artery disease.

We sought to investigate the association between change in leukocyte telomere length over 5 years and subsequent mortality in a prospective cohort study of in 608 patients with stable coronary heart disease (CHD).

## Methods

### Participants

The Heart and Soul Study is a prospective cohort study investigating the influence of psychosocial factors on cardiovascular events in patients with stable coronary artery disease. The enrollment process and methods have been described in detail previously [11] [13] [14]. Eligible participants were recruited from outpatient clinics in the San Francisco Bay Area if they met at least one of the following inclusion criteria: 1) history of myocardial infarction, 2) angiographic evidence of at least 50% stenosis by area in a least one coronary artery, 3) evidence of exercise-induced ischemia by treadmill electrocardiogram or stress nuclear perfusion imaging, or 4) history of coronary revascularization. Individuals were excluded if they had a history of myocardial infarction in the past 6 months, deemed themselves unable to walk 1 block, or if they were planning to move out of the local area within 3 years.

Between September 2000 and December 2002, a total of 1024 participants enrolled in the study. Of these, 954 provided DNA samples for analysis at the baseline visit, and 608 also provided DNA samples after 5 years of follow-up. The study protocol was approved by the Committee on Human Research at the University of California, San Francisco, and all participants provided written informed consent.

### Telomere Length Assay

Blood samples collected from participants at baseline and after 5 years of follow-up were subjected to density gradient centrifugation to yield a buffy coat preparation containing peripheral blood leukocytes and stored at -70C. Genomic DNA was isolated according to standard procedures using the Wizard Genomic DNA Purification Kit (Promega Corp, Madison WI). After verifying the integrity and purity of DNA, samples were diluted in 96-well microtiter source plates to a fixed concentration of 3 ng/ul. Relative mean telomere length was measured from DNA by a quantitative polymerase chain reaction (qPCR) assay that compares mean telomere repeat sequence copy number (T) to a reference single-copy gene copy number (S) in each sample as previously described and validated by comparison with Southern blot terminal restriction fragment (TRF) analysis[15]. Standard curves were derived from serially diluted reference DNA. The T/S ratio was determined from the average quantity of reference DNA found to match with each experimental sample for the copy number of the targeted template (the number of telomere repeats for T and the number of beta-globin gene copies for S). Measurements of baseline and 5-year DNA were performed at the same time; both baseline and 5 year samples from the same subjects were run on the same plate.

The primers for the telomere qPCR were tel1b [5’-CGGTTT(GTTTGG)5GTT-3’] and tel2b [5’-GGCTTG(CCTTAC)5CCT-3’], each used at a final concentration of 900 nM. Human beta-globin qPCR primers were: hbg1 [5’-GCTTCTGACACAACTGTGTTCACTAGC-3’], used at a final concentration of 300 nM, and hbg2 [5’-CACCAACTTCATCCACGTTCACC-3’], used at a final concentration of 700 nM. The final reaction mix was: 20 mM Tris-HCl, pH 8.4; 50 mM KCl; 200 nM each dNTP; 1% DMSO; 0.4xSybr Green I; 44 ng Escherichia Coli DNA; 0.8 U Platinum Taq DNA polymerase (Invitrogen) per 11 ul reaction; 10 ng genomic DNA. All PCRs were carried out on a Roche Lightcycler 480 real-time PCR machine (Roche Applied Science, Indianapolis, IN).

To control for inter-assay variability, eight control DNA samples were included in each run. The T/S ratio of each control DNA was divided by the average T/S ratio for the same DNA from each run to obtain a normalizing factor. The average normalizing factor was then used to adjust the participant DNA measurements to obtain the final T/S ratios in each batch. The coefficient of variability for the eight control samples across all batches was 6%. The T/S ratio at baseline and follow-up for each participant was measured in duplicate. When the duplicate T/S ratio and the initial value varied by more than 7%, the sample was run for a third time, and the two closest values were used. Approximately 15% of samples required assay in triplicate. Using this method, the inter-assay coefficient of variability for telomere length measurement was 3.7% (equivalent to 0.20 kilobases with respect to the baseline mean). The intra-assay coefficient of variability was 2.5% (equivalent to 0.13 kilobases with respect to the baseline mean).

To determine the conversion factor for the calculation of approximate base pair telomere length from T/S ratio, the above method was used to determine the T/S ratios, relative to the same reference DNA, for a set of genomic DNA samples from the human fibroblast primary cell line IMR90 at different population doublings, as well as with the telomerase protein subunit gene (hTERT) transfected into a lentiviral construct. The average T/S ratio for the Heart and Soul Study samples was lower than the range of T/S obtained with the IMR90 cell samples. The mean TRF length from the IMR90 samples was determined using Southern blot analysis, and the slope of the plot of mean TRF length versus T/S served as the conversion factor for calculation of telomere length in base pairs from the T/S ratio (S1 Fig). The equation for conversion from T/S ratio to base pairs for this study was base pairs = 3274+2413*(T/S).

### Other Measurements

All covariates were assessed at the 5-year examination. As previously described [11] [13] [14], demographics, age, sex, self-reported ethnicity, education, and income level were obtained by questionnaire. Cardiovascular co-morbidities and prior medical history were determined by self-report. Participants were weighed and measured without shoes. Body mass index was calculated as weight in kilograms divided by height in squared meters. Waist-to-hip ratio was calculated as waist circumference divided by hip circumference. Exercise capacity was measured at peak exertion during a symptom-limited exercise-treadmill stress test as previously described[13].

All patients underwent complete resting 2-dimensional echocardiography and Doppler examination using an Acuson Sequoia ultrasound system (Siemens Medical Solutions, Mountain View, CA) with a 3.5-MHz transducer. The left ventricular ejection fraction (LVEF) was calculated as (end diastolic volume—end systolic volume)/end diastolic volume. Fasting venous blood samples were obtained to measure serum biomarkers. HDL- and LDL-cholesterol levels and C-reactive protein (CRP) were measured in a clinical laboratory setting.

### Mortality

Following the 5-year examination, we conducted annual telephone interviews with participants or their proxies regarding recent emergency room visits, hospitalization or death. For any reported event, medical records, death certificates, and coroner’s reports were obtained and reviewed by 2 independent and blinded adjudicators. If the adjudicators agreed on the outcome classification, their classification was binding. If they disagreed, a third blinded adjudicator reviewed the event and determined the outcome classification.

All-cause mortality was determined by review of death certificates. The outcome variable was time from the 5-year examination to death or last follow-up. The mean length of follow-up after the 5-year examination was 4.2 ± 1.4 years (total duration of follow-up for Heart and Soul Study was 9.0 ± 1.4 years). Ascertainment of outcomes was achieved in 100% of participants.

### Statistical Analyses

Baseline and follow-up telomere lengths were normally distributed. To evaluate telomere change as a continuous variable, we regressed 5-year change on baseline telomere length, saved the residual, and evaluated the residual as the independent variable. To explore the relationship of this continuous variable with mortality, we graphed findings using deciles. To evaluate telomere length categorically, defined by trajectory, as done previously[12, 14] we divided leukocyte telomere trajectory into tertiles and categorized participants as shortened (lowest tertile), maintained (middle tertile) or lengthened (highest tertile). This resulted in the maintenance group including zero change, from -167 to +113, the shortened group ranged from -1012 to -169bp, and the lengthened group ranged from +114 to +1245bp.

Differences in participant characteristics were compared with the use of ANOVA for continuous variables and the chi-squared test for dichotomous variables, using the categorical trajectory variable. We generated a Kaplan Meier figure and used a log rank test to compare survival across the 3 categories. We then used Cox proportional hazard models to examine the association between telomere trajectory and all-cause mortality, adjusted for patient characteristics that differed between the trajectory groups (at p<0.10). Covariates were added sequentially in multivariable models to assess the incremental attenuation attributable to each adjustment step. Telomere trajectory was evaluated both as a categorical variable and as a continuous variable. To explore potential effect modifiers, we tested for interactions of telomere change with age, sex, and smoking in the final multivariable-adjusted model (all nonsignificant).

Since we have previously demonstrated that short baseline telomere length is a strong predictor of mortality[11], we sought to evaluate the incremental prognostic value of 5-year change in telomere length vs. a one-time measurement of telomere length. To determine the incremental prognostic value of calculating change in telomere length during the previous 5 years (vs. a single measure of telomere length at the 5-year exam), we calculated C statistics for models predicting mortality that were adjusted for each of these variables separately and estimated 95% confidence intervals from 5,000 bootstrap samples with replacement. Statistical analysis was performed using Intercooled Stata, version 9.2 (Stata Corp, College Station, TX).

## Results

The characteristics of the study population categorized by 5-year change in telomere length are shown in Table 1. After dividing the sample into tertiles, there were a total of 203 participants who experienced telomere shortening, 203 who maintained their telomere length and 202 who experienced telomere lengthening. As compared with participants who experienced telomere lengthening, those with telomere shortening were older and more likely to be male. Participants with telomere shortening also had higher waist to hip ratio, lower treadmill exercise capacity, higher LV ejection fraction, and worse kidney function. Baseline telomere length did not differ across the 3 categories, but those who experienced 5-year telomere shortening had shorter 5-year telomere length than those who experienced 5-year telomere lengthening. There were no differences in ethnicity, education, income, body mass index, or comorbidities across categories of telomere trajectory.

**Table 1:** Characteristics of participants by change in telomere length during the previous 5 years*.

	5-Year Change in Leukocyte Telomere Length			
Variable	Shortened	Maintained	Lengthened	P value
	N = 203	N = 203	N = 202	
Range (base pairs)	-1012 to -169	-167 to 113	114 to 1245	
Average base pairs/year	-202 to -34	-33 to 22.6	22.8 to 249	
Age, yrs	69 ± 9.5	66 ± 9.9	63 ± 10.2	<0.001
Male	184 (91)	167 (82)	148 (73)	<0.001
White	125 (62)	124 (61)	113 (56)	0.249
Income <$20,000	84 (42)	93 (46)	96 (48)	0.230
High school education	174 (86)	176 (87)	184 (91)	0.125
BMI, kg/m2	28.3 ± 4.6	28.9 ± 5.4	28.6 ± 5.4	0.499
Waist-hip ratio	0.98 ± 0.08	0.94 ± 0.08	0.94 ± 0.08	<0.001
Current smoking	34 (17)	31 (15)	36 (18)	0.756
History of HTN	144 (71)	144 (71)	137 (68)	0.496
History of MI	111 (55)	100 (49)	106 (52)	0.582
History of Stroke	33 (16)	20 (10)	22 (11)	0.100
History of HF	35 (17)	27 (13)	28 (14)	0.370
History of DM	52 (26)	48 (24)	46 (23)	0.504
Systolic blood pressure, mmHg	132 ± 19	133 ± 21	133.05 ± 20	0.630
Exercise capacity, METS	7.49 ± 2.96	8.10 ± 3.41	8.32 ± 3.23	0.012
LV ejection fraction	63 ± 8	63 ± 10	62 ± 9	0.062
HDL, mg/dl	45 ± 12	46 ± 15	47 ± 15	0.109
LDL, mg/dl	102 ± 33	108 ± 36	102 ± 31	0.916
C-reactive protein, mg/L	3.2 ± 4.0	4.2 ± 7.1	4.3 ± 8.2	0.100
Serum creatinine	1.2 ± 0.8	1.0 ± 0.4	1.1 ± 0.5	0.077
Baseline telomere length (bp)	5562 ± 577	5390 ± 523	5536 ± 465	0.609
5-year telomere length (bp)	4971 ± 204	5230 ± 165	5661 ± 260	<0.001

*Number (%) or mean ± SD

**Fig 1:**
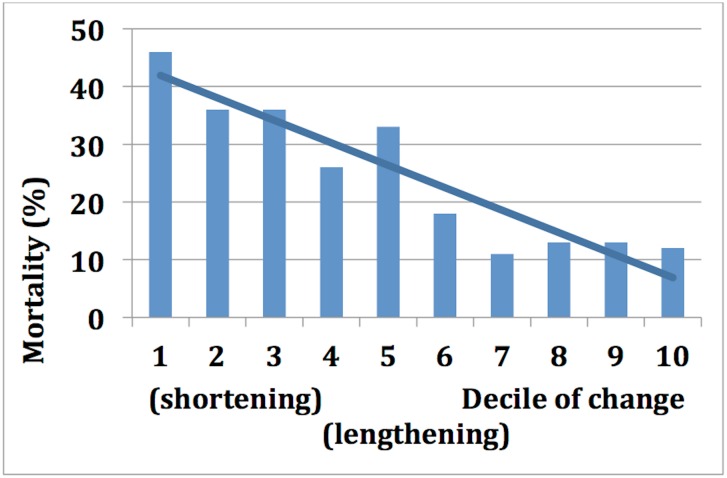
Mortality by decile of 5-year change in telomere length (p for trend <0.001).

During a mean follow-up of 4.2 ± 1.4 years, there were 149 deaths. Telomere change was inversely associated with mortality (Fig 1, Table 2). Mortality ranged from 46% in the lowest decile to 12% in the highest decile of change. In an unadjusted analysis, each standard deviation (325 base pairs) increase in telomere change was associated with a 36% lower risk of death (Table 3, HR 0.64, 95% CI, 0.54–0.76; p<0.001). After adjustment for age, sex, waist to hip ratio, exercise capacity, LV ejection fraction, serum creatinine, and year 5 telomere length, each SD increase in telomere change remained associated with a 24% lower risk of death (HR 0.76, 95% CI 0.61–0.94; p = 0.01).

**Table 2:** Mortality by decile of 5-year telomere change.

Decile	Range (base pairs)	Average base pairs/year	Number of study participants	Number (%)who died
**1**	-1012 to -371	-202 to -74	61	28 (46%)
**2**	-370 to -275	-74 to -55	61	22 (36%)
**3**	-274 to -184	-55 to -37	61	22 (36%)
**4**	-184 to -102	-37 to -20	61	16 (26%)
**5**	-99 to -32	-20 to -6	60	20 (33%)
**6**	-32 to 54	-6 to 11	61	11 (18%)
**7**	56 to 148	11 to 30	61	7 (11%)
**8**	148 to 268	30 to 54	61	8 (13%)
**9**	279 to 429	56 to 86	61	8 (13%)
**10**	430 to 1245	86 to 249	60	7 (12%)

**Table 3:** Association between 5-year telomere change [entered as a continuous variable, per standard deviation (325 base pair) increase] and mortality in 608 study participants.

	Hazard Ratio (95% CI)	P value
Unadjusted	0.64 (0.54–0.76)	<0.001
Model 1	0.74 (0.61–0.88)	0.001
Model 2	0.77 (0.64–0.91)	0.003
Model 3	0.76 (0.61–0.94)	0.01

Model 1 = adjusted for age, sex

Model 2 = adjusted for age, sex, HDL, and smoking

Model 3 = adjusted for age, sex, waist to hip ratio, exercise capacity, LV ejection fraction, serum creatinine, and year 5 telomere length

**Fig 2:**
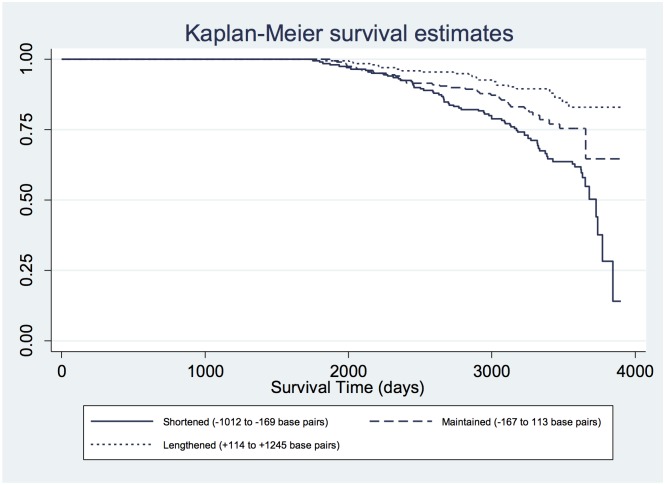
Survival in 608 patients with coronary heart disease, stratified by change in telomere length during the previous 5 years (overall log rank test p<0.0001).

Mortality occurred in 39% (79/203) of patients who experienced telomere shortening, 22% (45/203) of patients whose telomere length was maintained, and 12% (25/202) of patients who experienced telomere lengthening (p<0.001). As compared with patients who maintained telomere length, patients who experienced telomere lengthening had a 56% lower risk of mortality (Fig 2, Table 4, adjusted HR 0.44, 95% CI, 0.23–0.87). Mortality was higher in patients with telomere shortening (vs. maintenance), but this association was not statistically significant (HR 1.32, 95% CI, 0.84–2.08; p = 0.23). We found no evidence for an interaction of telomere category with age, sex or smoking (all p values for interaction > 0.05).

**Table 4:** Association between category of 5-year telomere trajectory and mortality.

	Shortened		Maintained	Lengthened	
	N = 203		N = 203	N = 202	
	HR (95% CI)	P value		HR (95% CI)	P value
**Unadjusted**	1.62 (1.12–2.34)	0.01	reference	0.53 (0.33–0.87)	0.01
**Model 1**	1.52 (0.99–2.32)	0.05		0.50 (0.28–0.90)	0.02
**Model 2**	1.33 (0.87–2.04)	0.19		0.59 (0.33–1.07)	0.09
**Model 3**	1.32 (0.84–2.08)	0.23		0.44 (0.23–0.87)	0.02

Model 1 = adjusted for Y5 telomere length

Model 2 = adjusted for age, sex and Y5 telomere length

Model 3 = adjusted for age, sex, year 5 telomere length, waist to hip ratio, exercise capacity, LV ejection fraction and creatinine

We calculated C statistics to compare the prognostic value of 5-year telomere change vs. a one-time measurement of telomere length for predicting mortality. After adjustment for age, sex, waist to hip ratio, exercise capacity, LV ejection fraction, and creatinine, the C statistic was 0.73 for a model with telomere change alone, 0.73 for a one-time measurement of telomere length (at year 5), and 0.69 for a model with both 5-year telomere change and a one-time measurement of telomere length (at year 5). There was no statistical difference between these C statistics.

## Discussion

Telomere maintenance has a significant impact on the ability of cells to continue dividing[16]. However, previous studies evaluating a single measurement of leukocyte telomere length as a predictor of mortality have yielded mixed results[17, 18]. We hypothesized that change in telomere length, when examined several years apart, may be a more robust indicator of telomere health (and a stronger predictor of mortality) than a one-time measurement. In this longitudinal study of 608 patients with stable coronary artery disease who were followed for 9.2 years, we found that change in leukocyte telomere length during the first five years was an independent predictor of mortality during the subsequent 4.2 years. Each 325 base pair increase in telomere length during the first 5 years was associated with 24% lower mortality during the subsequent 4.2 years. This observation raises the possibility that change in telomere length may influence mortality and/or serve as a biomarker of cellular aging.

The mechanism by which leukocyte telomere shortening and mortality are linked is not well understood. Traditional clinical risk factors do not seem to explain our finding, given the similarity between patients across a broad array of demographic, clinical, and biochemical variables. Biochemical stress, mediated via oxidative stress and inflammation, may provide a potential link between telomere shortening and mortality, as it is a known driver of telomere shortening, cardiovascular disease, and organismal aging[19, 20]. Oxidative stress has a direct negative effect on telomere length maintenance, both through inhibition of telomerase activity[21] and direct damage to GGG triplets in telomeric DNA[22]. Several diseases such as cardiovascular disease and malignancy have been linked to chronic oxidative stress and accelerated telomere shortening[23, 24].

Another potential mechanism for the link between telomere shortening and mortality may be in the activity of the enzyme telomerase. Lin et al found that changes in leukocyte telomere length were associated with telomerase activity [25]. Change in telomere length is regulated by many factors, including the activity of telomerase, a cellular ribonucleoprotein reverse transcriptase enzyme that maintains telomeric DNA, and cell division capacity. Both in vitro and human studies have shown that leukocyte telomeres can lengthen[26] [14]. Telomerase counteracts telomere shortening by addition of DNA nucleotides onto telomeres[27, 28]. In patients with cardiovascular disease, telomerase is crucial for cardiovascular cell functioning[29] and its deficiency has been linked to cardiovascular risk factors in vivo[30].

In addition, leukocyte telomere shortening may contribute directly to disease and mortality. As discussed, short telomeres lead to cell senescence and death [31, 32]. Senescent cells secrete pro-inflammatory cytokines[33], which can contribute to a pro-inflammatory milieu and resultant cellular damage.

Among the strengths of the present study are the detailed measurement of multiple potential confounding variables including comorbid conditions and cardiac disease severity. This study design allowed us to prospectively investigate the prognostic value of change in leukocyte telomere length in a large cohort of comprehensively phenotyped patients with CAD. However, several limitations must be considered when interpreting our results. First, our participants were mostly urban men, all with coronary artery disease, so our results may not generalize to other populations. Second, due to long term storage of blood samples, we were unable to measure biomarkers of oxidative stress or telomerase activity. Third, the question of whether telomere shortening is merely an epiphenomenon, or whether it plays an active role in mortality, cannot be answered on the basis of our results. However, the marked lack of variation between groups in terms of many clinical and laboratory factors suggests that telomere shortening is an independent risk factor not captured by traditional models of risk. Finally, as with any repeated measurement of a continuous variable, the possibility of regression to the mean must be considered. However, this is very unlikely because patients were not selected on the basis of baseline telomere length, and the measurements of year 5 telomere length had less variance than the baseline measurements [14].

In summary, we report that leukocyte telomere shortening over 5 years in a cohort of individuals with stable coronary artery disease is associated with subsequent mortality, independent of existing cardiac risk factors. Further studies should be aimed at investigating the mechanisms and significance of the association between change in telomere length and mortality in patients with stable CAD, as well as investigating whether these results can be replicated in other cohorts of patients.

## Supporting Information

S1 Fig Conversion of T/S ratios to base pairs. (JPG)

S2 Fig Telomere length by age in 608 participants. a. Upper left: Telomere length at baseline exam by age at year 6 (p<0.001). b. Upper right: Telomere length at year 6 by age at year 6 (p<0.001). c. Lower left: Crude change in telomere length by age at year 6 (p = 0.10). d. Lower right: Change in telomere length adjusted for baseline telomere length by age at year 6 (p<0.001).
(JPG)

S3 Fig Distribution of leukocyte telomere length at baseline and after 5 years of follow-up (reproduced from Farzenah-Far, *PLOS One* 2010). (JPG)
