# Correction: Change in Leukocyte Telomere Length Predicts Mortality in Patients with Stable Coronary Heart Disease from the Heart and Soul Study

**DOI:** 10.1371/journal.pone.0168868

**Published:** 2016-12-19

**Authors:** Sarah E. Goglin, Ramin Farzaneh-Far, Elissa S. Epel, Jue Lin, Elizabeth H. Blackburn, Mary A. Whooley

Fig 1 appears incorrectly in the published article. The x-axis label should display “(shortening)” on the left and “(lengthening)” on the right. Please see the corrected Fig 1 here.

**Fig 1:**
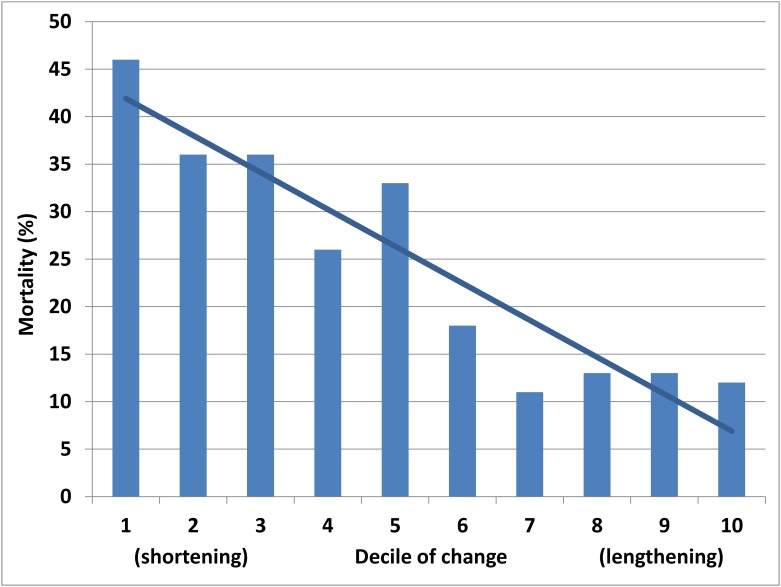
Mortality by decile of 5-year change in telomere length (p for trend <0.001).

## References

[1] Goglin SE, Farzaneh-Far R, Epel ES, Lin J, Blackburn EH, Whooley MA (2016) Change in Leukocyte Telomere Length Predicts Mortality in Patients with Stable Coronary Heart Disease from the Heart and Soul Study. PLoS ONE 11(10): e0160748 doi: 10.1371/journal.pone.0160748 27783614 10.1371/journal.pone.0160748 PMC5081189

