# Supplementary figures and images for: Change in Leukocyte Telomere Length Predicts Mortality in Patients with Stable Coronary Heart Disease from the Heart and Soul Study
Source: PLoS One. 2016 Oct 26;11(10):e0160748. doi: 10.1371/journal.pone.0160748 (PMC5081189; doi:10.1371/journal.pone.0160748)

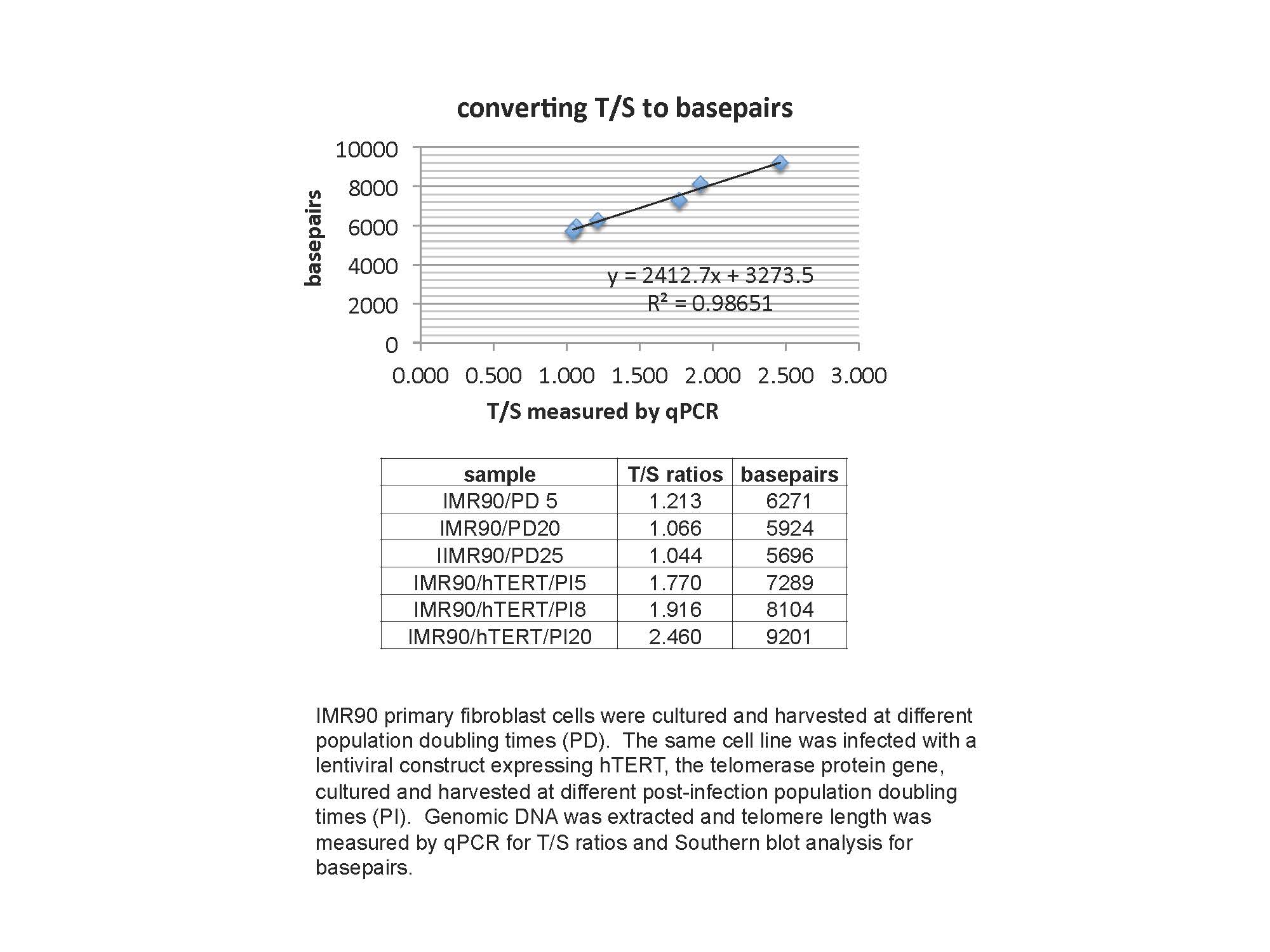

Supplement: S1 Fig — (JPG) [file pone.0160748.s001.jpg]

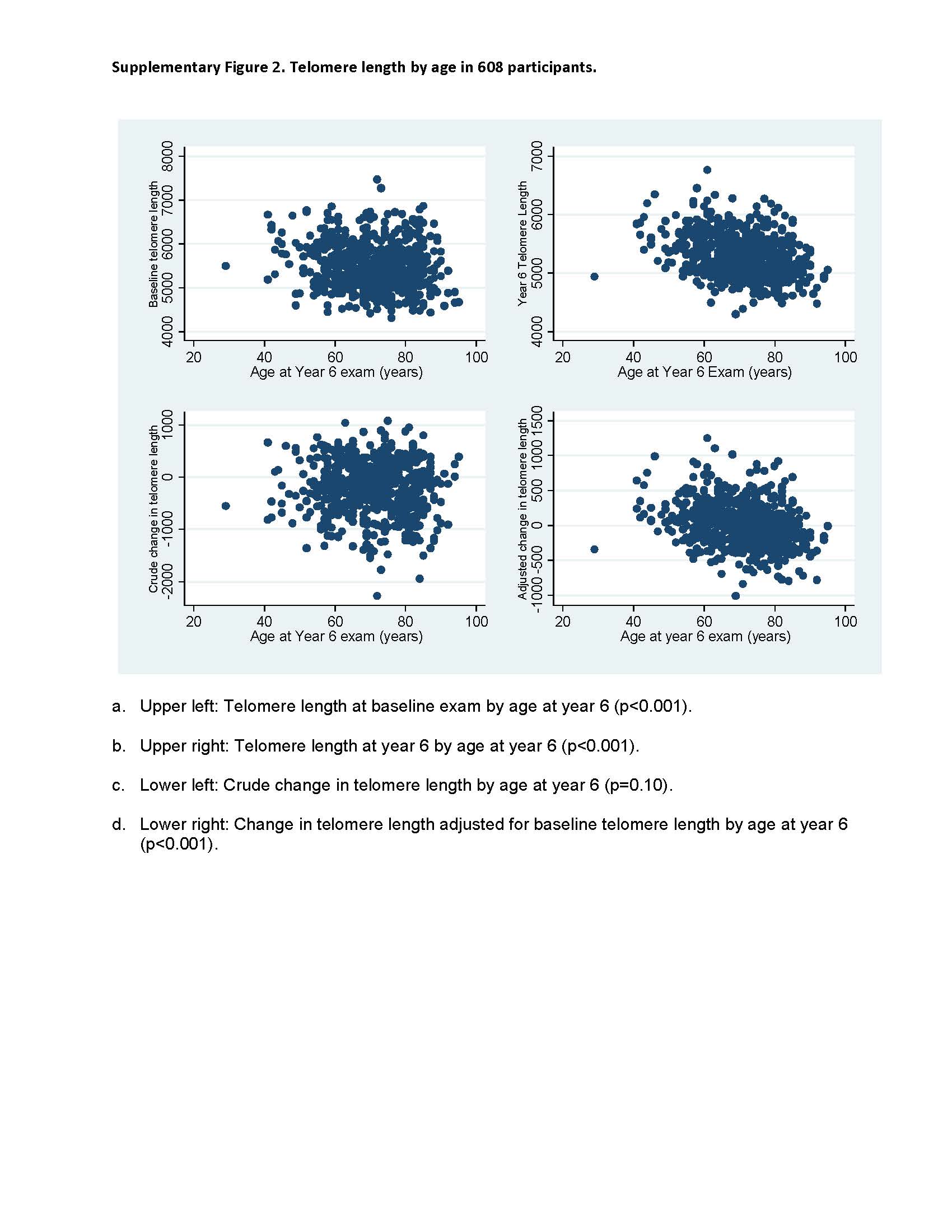

Supplement: S2 Fig — a. Upper left: Telomere length at baseline exam by age at year 6 (p<0.001). b. Upper right: Telomere length at year 6 by age at year 6 (p<0.001). c. Lower left: Crude change in telomere length by age at year 6 (p = 0.10). d. Lower right: Change in telomere length adjusted for baseline telomere length by age at year 6 (p<0.001). (JPG) [file pone.0160748.s002.jpg]

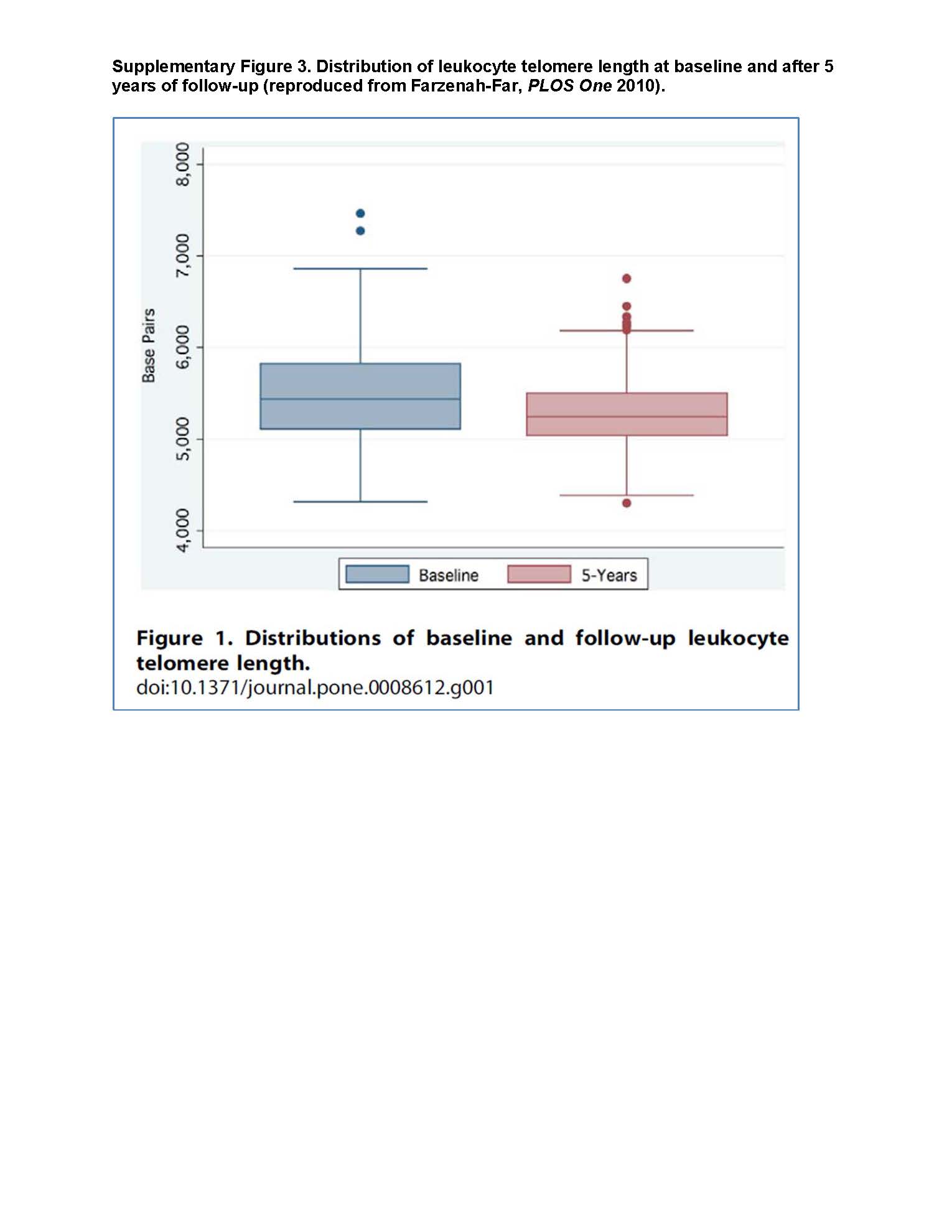

Supplement: S3 Fig — (JPG) [file pone.0160748.s003.jpg]
